# Using Adhesive Glue to Repair First Degree Perineal Tears: A Prospective Randomized Controlled Trial

**DOI:** 10.1155/2014/526590

**Published:** 2014-06-26

**Authors:** Tomer Feigenberg, Esther Maor-Sagie, Einat Zivi, Mushira Abu-Dia, Assaf Ben-Meir, Hen Y. Sela, Yossef Ezra

**Affiliations:** ^1^Department of Obstetrics and Gynecology, Hadassah-Hebrew University Medical Center, Ein-Kerem, Jerusalem, Israel; ^2^Department of Obstetrics and Gynecology, Trillium Health Partners, 2200 Eglinton Avenue W, Mississauga, ON, Canada L5M 2N1

## Abstract

Our objective was to evaluate the effectiveness of adhesive glue in repairing first degree perineal tears. We conducted a noninferiority prospective, randomized, controlled trial comparing adhesive glue with traditional suturing. Each case was evaluated immediately after birth and after the puerperium. The two-sample *t*-test and the Mann-Whitney nonparametric test were applied to compare quantitative variables between the treatment groups. The chi-squared test and Fisher's exact test were used to assess the association between qualitative variables. A total of 102 women participated, 28 in the suture arm and 74 in the adhesive glue arm. While cosmetic and functional results of adhesive glue use were not inferior to suturing, the use of adhesive glue was associated with a shorter procedure, less need for local anesthetic, less pain, and greater satisfaction. Our results suggest a novel approach for the repair of common postpartum first degree lacerations. The use of adhesive glue achieves cosmetic and functional results equal to traditional suturing and offers some immediate advantages for the patient. While further clinical trials are needed to validate our results, it is important to inform obstetrician of the possible use of adhesive glue in these very common clinical scenarios. This trial is registered with NCT00746707.

## 1. Introduction

Perineal trauma occurs in 85% of women after having a vaginal birth. It is estimated that 60–70% of these lacerations will be surgically repaired [1]. Sutured spontaneous tears are reported in approximately one-third of women in the United States of America [2] and the United Kingdom [3]; however, this is likely an underestimate. Perineal lacerations are classified according to the depth of the wound and the number of tissue layers involved. First degree perineal tears are defined as tears involving the skin, with no involvement of the muscular layer. The “gold standard” method for repairing perineal tears is to use absorbable (preferably fast-absorbing) sutures. A systemic review of the literature, conducted in 2008, has revealed that nonsuturing of perineal skin alone in first and second degree tears and episiotomies may reduce pain and dyspareunia in the puerperium but may lead to increased risk of wound gaping [3]. The ideal method for perineal laceration repair should be quick, painless, and easy to perform and preferably will not increase pain and dyspareunia during the puerperium.

Synthetic cyanoacrylate adhesives are a family of liquid monomers which polymerize at room temperature in an exothermic reaction (releasing heat in the process) on contact with a small amount of water or basic fluid to form polymers. They form strong adhesive bonds with a variety of substrates such as soft tissue [4]. On 26 August, 1998, FDA approved the first cyanoacrylate tissue adhesive device for topical skin approximation-Dermabond. Since then, the FDA has approved 2 more cyanoacrylate tissue adhesives for topical skin approximation including Histoacryl. In general, changes in the alkyl side chain (-R) of the cyanoacrylate adhesive will determine its properties such as the rate of degradation, rate of polymerization (with release of heat in the process), toxicity, flexibility, and the properties of the adhesive formed. The polymerized monomers are designed to slough off from the skin as the wound reepithelializes usually within 5–10 days. Tissue adhesive glues have been used for many years to treat small wounds and cuts. Various recent studies have shown that adhesive glues can be successfully applied to different mucosa and skin, as in the treatment of aphthous ulcers [5], lip closure [6], facial wounds after Mohs surgery [7], and nail bed injuries [8]. Other studies have shown successful use of adhesive glues in obstetrics and gynecology such as skin closure of Pfannenstiel incision at the end of cesarean section and repair of evulsion of clitoris [9, 10]. The fact that adhesive glues can be used in areas that resemble the vaginal mucosa has led us to believe that adhesive glues may be useful to treat superficial postpartum perineal tears.

The aim of this noninferiority prospective, randomized, controlled trial was to evaluate the use of two types of adhesive glue, octyl-2-cyanoacrylate (Dermabond, Ethicon) and n-butyl-2-cyanoacrylate (Histoacryl, B Braun), to treat first degree perineal tears, and to compare tissue gluing with a traditional suturing procedure using Vicryl Rapide fast-absorbing running sutures.

## 2. Materials and Methods

The primary outcomes of this study were cosmetic and functional effects on the damaged area, on a 10-point scale, as assessed at least six weeks postpartum by a physician who was blinded to the method of repair. Our basic hypothesis was that adhesive glues are not inferior to conventional suturing in terms of the quality of repair; therefore, the study was designed to have a power of 99.9%.

The study was designed as a noninferiority study, assuming that the means of cosmetic and functional scores for suturing and adhesive glue populations were equal (assumed to be a mean score of 9 out of 10), with a common within-group standard deviation of 0.90, and that a difference of 2 points or less would be clinically unimportant. Hence, the sample sizes of the two groups were intended to be 25 in the suture arm and 70 in the adhesive glue arm (35 in each adhesive glue group), and alpha (single-tailed) was set at 0.05. Patients were recruited until the goal number for the second postpartum evaluation was reached.

Eligible for the study were all women over the age of 18, who were diagnosed with first degree perineal tear, following birth. Excluded were women with any signs of local infection in the area to be repaired, women who chronically use steroids, women with excessive bleeding that does not allow the use of glue, and those with known allergy to adhesive glues. Women were randomized using numbered sealed envelopes containing concealed allocation cards randomly inserted by one of the researchers (T.F).

Institutional review board approval was obtained prior to the initiation of the study. All participants provided their written, informed consent when they were enrolled in the study. To meet institutional review board's requirements, a pilot study of 15 patients, 5 patients in each group, was designed to assess the feasibility of glue use. These patients were included in the final analysis according to group assignment (adhesive glue versus suturing).

For patients being treated with adhesive glue, the area to be glued was washed with aqueous chlorhexidine. A small gauze pad was placed on the tear itself, and another gauze pad was placed in the vagina proximal to the tear to protect the area from blood and secretions. The gauze within the tear was removed, and either octyl-2-cyanoacrylate or n-butyl-2-cyanoacrylate glue was immediately applied to the manually approximated tear. A second layer of the same glue was applied to the exterior skin. No drapes or local anesthesia was used. For all patients who were sutured, drapes were used and local anesthesia was applied to patients who did not have effective regional anesthesia; we used Vicryl Rapide 2 × 0 running sutures in the vagina and interrupted sutures on the perineum.

Immediately after the procedure, each physician completed a questionnaire evaluating the duration of the procedure, the location length and depth of the tear, the physician's assessment of and satisfaction with the results, and the patient's pain analogue and satisfaction scales (each on a 10-point scale). All patients were required to attend a follow-up appointment consisting of a repeat evaluation by a doctor blinded to the method of repair, at least six weeks postpartum. At that appointment, the cosmetic and functional outcomes, perineal pain during the puerperium, and patient satisfaction with the method of repair were evaluated. Patients who had been sutured in the past were also asked about their preference between suturing and the use of adhesive glue.

To compare quantitative variables between the treatment groups, the two-sample* t*-test and the Mann-Whitney nonparametric test were applied. The chi-squared test and Fisher's exact test were used to assess the association between two qualitative variables. All applied tests were single-tailed, and a *P* value of 5% or less was considered statistically significant.

## 3. Results

**Figure 1:**
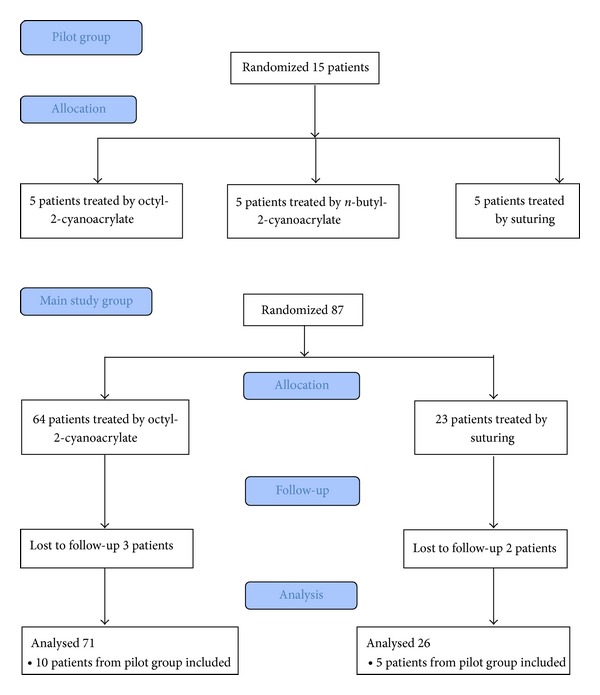
Patient's flow diagram.

We initiated the study with the recruitment of a pilot study of 15 patients, five in the octyl-2-cyanoacrylate group, five in the n-butyl-2-cyanoacrylate group, and five in the suturing group. Planed analysis of the pilot study has revealed that the use of n-butyl-2-cyanoacrylate resulted in unpleasant heat production when the glue came into contact with blood or secretions. It was subsequently decided to use only octyl-2-cyanoacrylate for all patients in the adhesive glue arm. The pilot study results were included in the final statistical analysis. The study was conducted between October 2009 and June 2011. At the end of the study, there were 28 patients in the suturing arm and 74 patients in the adhesive glue arm. Patient's flow diagram is presented in Figure 1. There was no difference between the two groups regarding mean age, parity, mean tear length as assessed by the physician performing the repair, or percentage of women who had epidural analgesia during labor and delivery. Although the mean tear depth was statistically shorter in the glue group, with a mean of 1.2 cm for the suturing arm versus 1.1 cm in the glue arm, we believe that this difference is clinically insignificant. No other differences were noted between the groups. The basic characteristics of the two groups are summarized in Table 1.

In three cases, we had to convert the use of adhesive glue to suturing due to failure of the adhesive glue (mainly because of bleeding). The results of these three cases were analyzed in an intent-to-treat manner. A total of 26 women from the suturing arm and 71 women in the adhesive glue arm completed the follow-up examination and evaluation. The scores for both cosmetic and functional results were high for the two groups. The mean cosmetic score was 9.4 for the suture group and 9.56 for the glue group. The mean functional score was 9.46 for the suture group and 9.73 for the glue group. These results demonstrate that the use of adhesive glue was not inferior to traditional suturing in the repair of first degree perineal tears.

Initial evaluation of each method of repair was conducted immediately after the procedure. The mean procedure time was significantly shorter for gluing than for suturing (2.29 versus 7.88 min). Pain sensation was also statistically lower in the adhesive group. The mean pain score was significantly higher among women who were sutured than among women who were glued (4.14 versus 1.72), despite the use of local anesthesia. There was no difference in doctor satisfaction score. We observed no differences between mucosa and skin in terms of successful application of glue. Results are presented in Table 2.

The results of the examination that was performed at least six weeks postpartum demonstrate that the duration of postpartum perineal pain was similarly short in both groups. During the puerperium, the mean pain level in the area of tissue damage did not differ significantly between the two groups, and patient satisfaction scores for the two methods were similarly high. Thirty-eight women in the glue arm had experienced suturing in one or more previous deliveries. When asked which method they would prefer for future perineal repair, 89.2% of these patients favored adhesive glue for future deliveries. Results of cosmetic and functional scoring are presented in Table 3.

## 4. Discussion

These results demonstrate that the use of adhesive glue (mainly octyl-2-cyanoacrylate tissue glue) for the repair of first degree perineal tears is not inferior to traditional suturing in terms of cosmetic and functional results. Both methods of repair resulted in excellent outcomes. We were able to demonstrate some potential advantages to the use of adhesive glue, including a shorter procedure time, and a substantial reduction in pain score (exempting the need for local anesthesia). We did not encounter any significant side effects from the use of adhesive glues for the treatment of postpartum laceration, such as contact dermatitis that has been previously reported [11, 12].

Adoni and Anteby [13] have compared the use of Histoacryl (n-butyl-2-cyanoacrylate) and suturing for episiotomy repair in 20 women. They found that n-butyl-2-cyanoacrylate was associated with less pain at the episiotomy site and less pain while walking, sitting, sleeping, lying down, nursing, urinating, and defecating. Rogerson et al. [14] used Indermil (which contains a similar composition of n-butyl-2-cyanoacrylate) to repair 20 second degree tears and episiotomies. As these were deeper tears, the first two layers were repaired routinely using Vicryl, suture for the vagina and deep layer to produce close approximation of the skin edges. While they experienced no immediate failed procedures, their results demonstrated that only 13 women (65%) completely healed. Interestingly, three women commented on a burning sensation during the application of the adhesive.

The authors concluded that n-butyl-2-cyanoacrylate is a safe and effective tool for the repair of such tears. Our experience supports previous reports that n-butyl-2-cyanoacrylate adhesive glues are associated with substantial heat production when they come into contact with body secretions or blood. By contrast, the use of the newer octyl-2-cyanoacrylate tissue glue is well tolerated and is not associated with such a phenomenon.

Our results support potential benefits of glue use, such as shorter procedure time and reduced pain sensation. Additionally, according to our experience, the use of glue is easy to learn (an untrained person can master the technique after up to 5 procedures). We have experienced only 3 cases of adhesive glue failure, all of which were related to excessive bleeding and required suturing at the time of the primary procedure. We did not experience any cases of delayed failure of the glue after the patient has left the delivery room. We believe that octyl-2-cyanoacrylate can be used to repair any perineal tear, as long as the tension on the glue is not excessive, especially when the edges do not require approximation. However, our study has a few limitations: mainly, its relative small size and the fact that it included first degree perineal tears only. In addition, we have no information comparing healing duration between the two groups as all our patients were seen at least 6 weeks postpartum at the time of routine follow-up; by that time, all lacerations were already completely healed. It is difficult to draw conclusions about the use of adhesive glues for the repair of more complicated perineal tear; thus, future studies are needed to determine whether octyl-2-cyanoacrylate can be used for second degree, third degree, and episiotomy repairs. As current octyl-2-cyanoacrylate application requires that the active glue liquid be squeezed through a sponge at the end of the applicator and then be used as a roller to apply the glue to the skin, a new appliance designed for specific perineal use would be beneficial.

In conclusion, octyl-2-cyanoacrylate tissue glue is an equal alternative for repair of first degree perineal tears. The use of octyl-2-cyanoacrylate is related to shorter procedure time and less pain, with functional and cosmetic results similar to those associated with conventional suturing. Future studies will determine its feasibility for second and third degree tears and episiotomy repair.

## Figures and Tables

**Table 1:** Basic characteristics of study's participants.

	Stitches mean (*N*)	Glue mean (*N*)	Statistical test	*P* (2-tailed)
Patient age	29.29 (28)	29.92 (74)	*t*-test	.59
Parity	2.46 (28)	2.68 (74)	*t*-test	.5
Length of tear, cm	1.7 (28)	1.9 (74)	*t*-test	.236
Depth of tear, cm	1.3 (26)	1.1 (71)	*t*-test	.047
Epidural	12/28	30/74	Chi-squared	.38

**Table 2:** Results of evaluation of perineal repair immediately after the procedure.

	Stitches mean (*N*)	Glue mean (*N*)	Statistical test	*P* (1-tailed)
Mean repair time, minutes	7.88 (26)	2.29 (67)	*t*-test	<.001
Patient satisfaction score (1–10)	9.42 (28)	9.42 (74)	*t*-test	.013
Patient pain score (1–10)	4.14 (28)	1.72 (72)	*t*-test	<.001
Doctor satisfaction score (1–10)	9.35 (23)	9.35 (68)	*t*-test	.494
Use of local anesthesia	18/27 (66%)	2/74 (2.7%)	Chi-squared	<.001

**Table 3:** Results of follow-up evaluation (six weeks or more after delivery).

	Stitches mean (*N*)	Glue mean (*N*)	Statistical test	*P* (1-tailed)
Cosmetic score (1–10)	9.4 (25)	9.56 (71)	*t*-test	.220
Functional score (1–10)	9.46 (26)	9.73 (71)	*t*-test	.071
Pain duration in the damaged area during puerperium, days	5.17 (24)	6.76 (68)	*t*-test	.229
Pain score in the damaged area during puerperium (1–10)	3.76 (25)	3.27 (70)	*t*-test	.240
Patient satisfaction score at follow-up (1–10)	8.55 (22)	9.42 (65)	*t*-test	.074
Proportion of patients that would prefer glue given past suture experience, *N* (%)	2/38 (5.2%)	33/38 (86%)	Chi-squared	<.001
